# Porosome in the Exocrine Pancreas: A Detailed EM Study suppressor

**DOI:** 10.15190/d.2014.15

**Published:** 2014-08-05

**Authors:** Constantin Craciun

**Affiliations:** Babes-Bolyai University, Cluj-Napoca, Romania

**Keywords:** Porosome Complex, Secretion, Exocrine Pancreas

## Abstract

A major question in cell biology that accumulation of partially empty vesicles in cells following secretion is seen, while it is believed that secretion occurs via the complete merger of secretory vesicles with the cell plasma membrane. This important question was solved nearly two decades ago, with the discovery of the Porosome. Porosomes are cup-shaped lipoprotein structures found at the plasma membrane of all cells. Secretory vesicles dock and transiently fuse at the porosome base to form a continuous channel or fusion pore to release the pressurized vesicle contents to the outside. In a decade-long study by our group, we carefully examined using electron microscopy, the detailed structure of the porosome complex in acinar cells of the exocrine pancreas. Besides conformation of earlier findings, our study provides in much greater detail, the in situ morphology of the porosome complex in the exocrine pancreas. The discovery of the detailed morphology of the exocrine pancreas porosome complex in my laboratory is one of the major highlights of my academic career spanning nearly 50 years. Results from our EM studies, reveal for the first time the presence of tethers or cables, which are likely t-SNAREs, present at the porosome base. These EM studies further demonstrate for the first time the docking of a single secretory vesicle or zymogen granule at the base of more than one porosome complex. Detailed spoke-like elements lining the porosome cup were also observed for the first time in these studies, greatly advancing our understanding of the molecular architecture and physiology of the porosome in the exocrine pancreas.

## INTRODUCTION

In the first January 1997 issue of the Proceedings of the National Academy of Sciences of the United States of America^1^, a breakthrough in our understanding of live cell structure-function at nanometer resolution, using a new technology of atomic force microscopy, was reported. This reported breakthrough was also a big discovery, since after nearly half a century, a new cellular structure ‘the porosome’ was discovered, and determined to participate in the important cellular process of secretion. More importantly, it solved a major question in cell biology, since the accumulation of partially empty vesicles in cells following secretion was previously unexplainable due to the belief that all secretion occurs via the complete merger of secretory vesicle membrane with the cell plasma membrane^2-23^. The porosome discovery provided the answer, demonstrating that secretory vesicles transiently fuse at the porosome base to establish a fusion pore or channel through which secretory products are released in a highly regulated manner from cells during secretion^24-36^. The excitement of the porosome discovery prompted several commentaries and articles on the subject^37-53^, the most note worthy among them being an editorial in the journal Pancreatology in 2004^40^ that followed a review article in the journal Science in 2003^43^. The editorial in Pancreatology entitled “Legacy of a Distinguished Scientist: George E. Palade”, states “After almost 50 years since the discovery of the ribosome by Prof. George E. Palade in the pancreas and other cell types, we are witnessing the discovery of another new cell structure.” “Following in the footsteps of his grand mentor, Prof. Bhanu Jena has also made equally pioneering contributions to cell physiology. Similar to his grand mentor, George E. Palade, B. Jena’s utilization of new nanotechnologies, such as the atomic force microscopy, in combination with conventional technologies like electron microscopy, biochemistry, and electrophysiology, brought understanding of the cell to a new next level. B. Jena and his research team have discovered a new cellular structure, the ‘porosome’, located at the cell plasma membrane, where secretory vesicles fuse to release their cargo. This work has revealed, at nanometer resolution, the molecular structure and dynamics of the ‘porosome’ or fusion pore in live cells. The biochemical composition of the ‘porosome’ and its functional reconstitution into artificial lipid membranes have also been found. B. Jena and his group also determined the molecular mechanism of fusion of the secretory vesicle membrane with the ‘porosome’ membrane and the regulated expulsion of intravesicular contents. These pioneering findings have given birth to yet another important field in cell biology, nano cell biology.”

**Figure 1:**
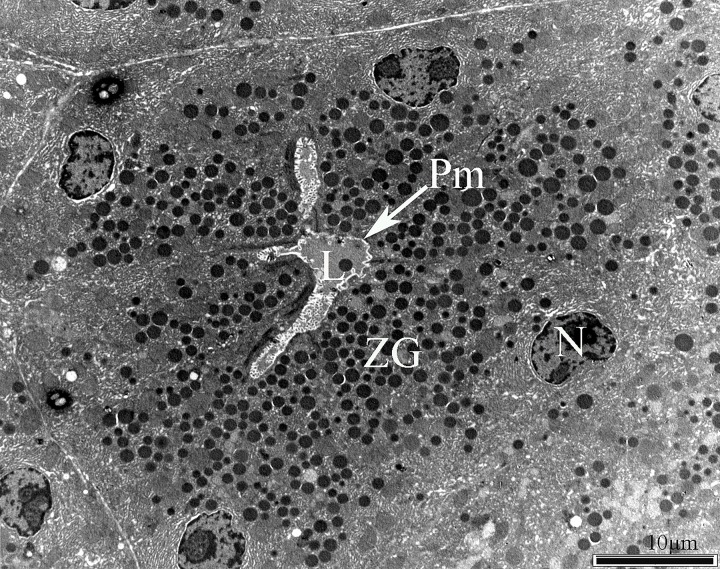
Electron micrograph of acinar cells of the exocrine pancreas, demonstrating the acinar lumen (L), the plasma membrane (Pm), and the basolaterally located nucleus (N).

**Figure 2:**
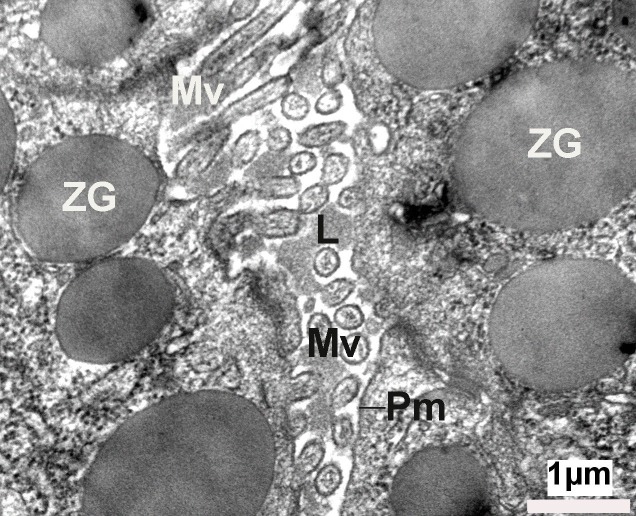
Electron micrograph of a close up view of the apical end of pancreatic acinar cells, demonstrating the presence of the lumen (L), microvilli (Mv), and the plasma membrane (Pm). Note the apically located electron-dense zymogen granules (ZG).

The 100-180 nanometer in size porosome complex in the exocrine pancreas and in growth hormone secreting cells of the pituitary gland, and the 10-17 nanometer porosomes at the nerve terminal and in astrocytes, are difficult to be sectioned through for electron microscopy, and therefore had not been discovered earlier. My decades of experience in electron microscopy, and the visits by Professor Palade to my laboratory and his continued encouragement, prompted me to initiate studies in 2003 on the porosome complex in the exocrine pancreas. After a decade of careful study using electron microscopy, our results were published in 2013^34^. Results from our EM studies reveal for the first time the presence of tethers or cables, which are likely t-SNAREs, present at the porosome base. These TEM studies further demonstrate for the first time the docking of a single secretory vesicle or zymogen granule at the base of more than one porosome complex. Detailed spoke-like elements lining the porosome cup were also observed for the first time in these studies, greatly advancing our understanding of the molecular architecture and physiology of the porosome in the exocrine pancreas.

**Figure 3:**
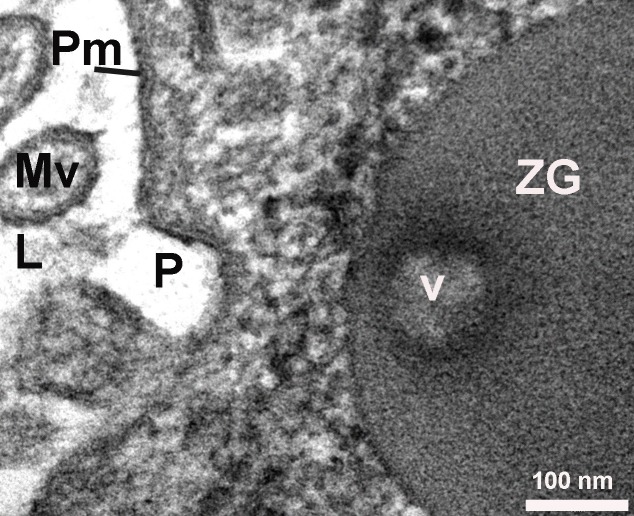
High-resolution electron micrograph of a cup-shaped porosome (P) at the cell plasma membrane, and the imprints (V) of the porosome left on a zymogen granule (ZG) that had docked at the porosome base. Mv-Microvili.

**Figure 4:**
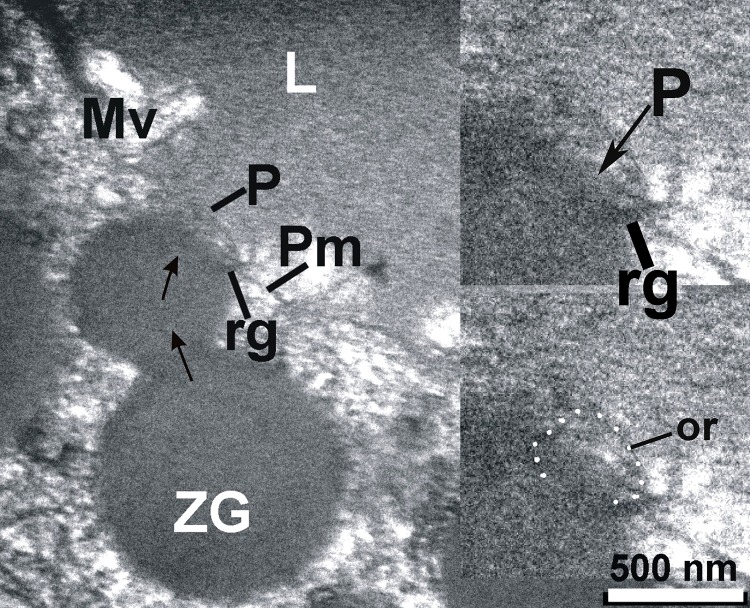
ZG found near plasma membrane (Pm) that delimit the lumen (L) of acinus, is in contact with a P to deliver their content in lumen; it has smaller diameter after partially delivery of their content; the outer ring of porosome (or) and lateral ridges (rg) are visible in inset.

## STRUCTURE OF POROSOMES IN THE EXOCRINE PANCREAS

**Figure 5:**
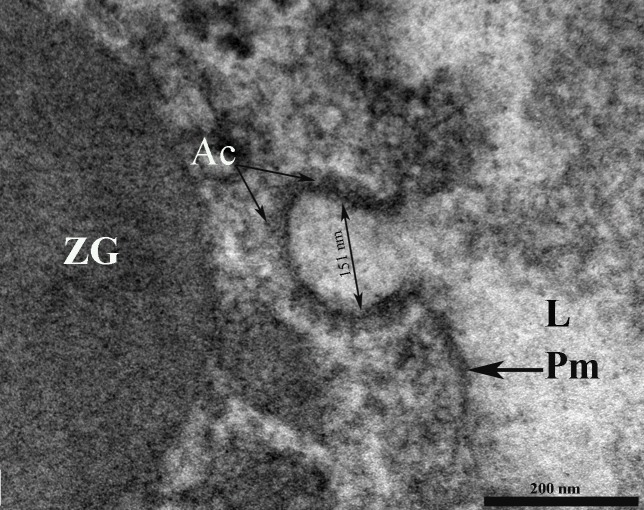
Porosome base (P) containing anchoring cables (Ac) or tethers measuring 15 – 20 nm in length. Porosome diameter has 151 nm. ZG – zymogen granule; L – lumen; Pm – plasma membrane.

**Figure 6:**
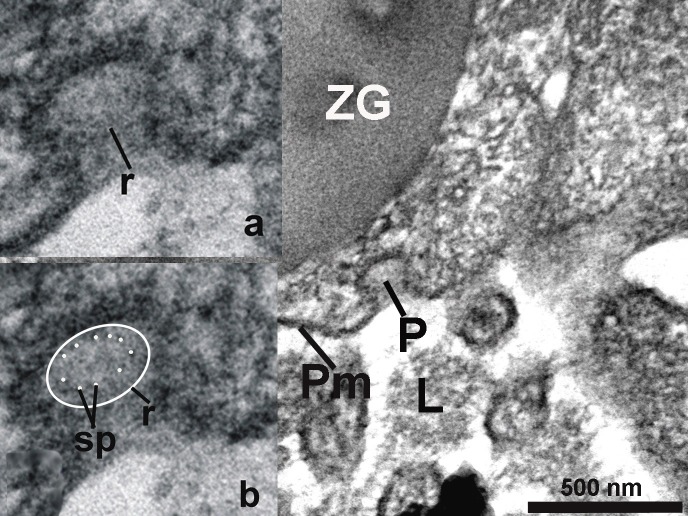
The porosome (P) with 10 fine filaments (spokes – sp) surrounded by a ring (r) are visible in cros section of the porosome (see insets a and b). L - lumen; Pm – plasma membrane; ZG – zymogen granule.

**Figure 7:**
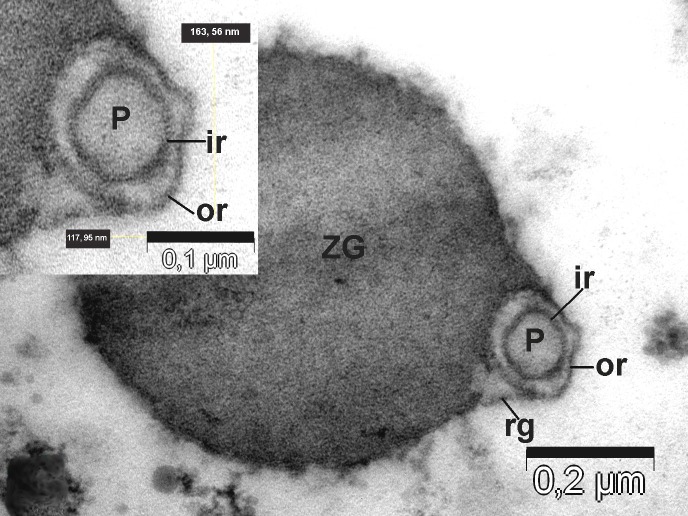
Top view of the porosomes (P) associated (co-located) with zymogen granules (ZG) obtained from the stimulated tissue using 1 µM of the secretagogue carbamylcholine. Transverse section showing two rings of porosomal complex. ir – inner ring; or – outer ring; rg – ridges.

**Figure 8:**
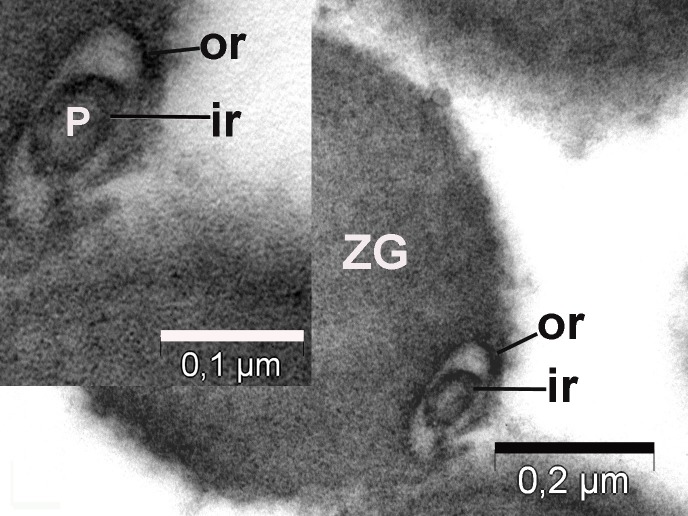
Top view of the porosomes (P) associated (co-located) with zymogen granules (ZG) obtained from the stimulated tissue using 1 µM of the secretagogue carbamylcholine. Transverse section showing two rings of porosomal complex. ir – inner ring; or – outer ring; rg – ridges.

**Figure 9:**
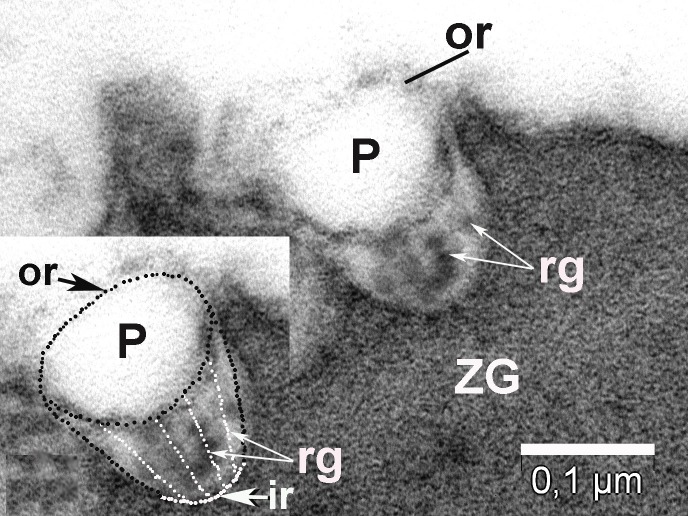
The porosome (P) has an inner ring (ir) of 60 nm, an outer ring (or) of 176 nm diameter and ten lateral-vertical ridges (rg) that link these two rings. ZG – zymogen granule.

The exocrine pancreas is composed of acini with polarized pyramidal acinar cells, with the basolateral region containing the nucleus and many zymogen granules (ZG) in cytoplasm. At the apical membrane of the cells, microvilli and porosomes are present (**Figure 1**, **Figure 2**). Zymogen granules, the secretory vesicles in exocrine pancreas, are known to dock at the apical plasma membrane of acinar cells, to release their contents to the outside (**Figure 2**). A careful and detailed analysis of the apical end of pancreatic acinar cells (**Figure 3**, **Figure 4**, **Figure 5**, **Figure 6**, **Figure 7**, **Figure 8**, **Figure 9**) clearly demonstrates the presence of approximately 100 nm in diameter microvilli (**Figure 2**, **Figure 3**), and the presence of 80-200 nm cup-shaped porosomes, some having docked zymogen granules. Although it has been suggested that a series of ZG could fuse resulting in compound fusion, this type of fusion between large granules is an extremely rare event, and if observed, it is between small and large vesicles, suggesting the possible replenishment of partially spent ZG following stimulation of cell secretion (**Figure 4**).

On close examination of porosomes, anchoring cables or tethers measuring 15-20 nm in length are sometime clearly observed at the porosome base (**Figure 5**). t-SNAREs and calcium channels had previously been reported and demonstrated to be present at the base of the pancreatic porosome complex^27,53^, suggests tethers to be t-SNAREs. However, since these tethers appear to be larger than SNAREs, and appear to be present at the lateral and basal regions of the porosome cup, suggests them to represent cytoskeletal and motor proteins, possibly composed of actin, vimentin, fodrin, and myosin, as previously reported^27^, and which have also been confirmed using mass spectrometry^33^. Since our study was carried out in cells following stimulation of secretion^34^, we were able to observe the base of the porosome structure post docking as well as the impression of the portion of the vesicle that had just transiently docked at the porosome base (**Figure 6**). Ring of fine filamentous structures are clearly visible within the porosome complex^34^. Also for the first time, we were able to demonstrate the interaction of a single ZG with more than one porosome complex^34^. This scenario had been previously suggested, however never observed. Unlike in neurons where literally hundreds of 10-17 nm porosomes are present at the presynaptic membrane, just a dozen such structures may be present at the apical end of a pancreatic acinar cell, hence the difficulty in observing them.

Furthermore, depending on where and at which angle the section is taken, it is very difficult to observe a detailed structure of the pancreatic acinar cell porosome complex.

To further understand the porosome morphology and its interactions with ZG, the exocrine pancreas was stimulated using 1 µm of the secretagogue carbamylcholine, followed by isolation of ZG, their mild fixation, and electron microscopy. Our study^34^ clearly demonstrated the co-isolation of porosomes with docked ZG, providing a further understanding of the porosome structure-function and physiology. Careful study of the TEM micrographs of isolated ZG demonstrated the cup-shaped morphology of the porosome complex as previously reported. Isolated ZG exhibit in their periphery cup-shaped structures of diameters ranging from 152 – 162 nm, similar to size of the porosome complex. Similarly, top view of porosomes associated with ZG demonstrate the presence of two rings, inner ring measuring approximately 60 nm, and a 176 nm outer ring^34^ (**Figure 7**, **Figure 8**). Some ridges are also seen originating from the inner ring toward the outer ring, suggesting the linking of these two rings to form a basket-like morphology (**Figure 9**). Such structures were previously demonstrated in isolated membrane-free porosome complexes^27,28^. Another important finding was the complete porosome cup associated with the ZG and its detailed structure. The diameter at the top of cup-shaped porosome measure approximately 140–150 nm, and at its base 30–40 nm. When porosomes were observed in vertical sections, they appear to have two concentric rings, delimitated by the lateral ridges (**Figure 9**).

## CONCLUSIONS

Results from our study^34^demonstrated for the first time the presence of tethers, and are associated at the base of porosomes. At times, such tethers are seen to connect ZG to the porosome base. Furthermore, for the first time our studies demonstrate the docking of a single secretory vesicle to the base of more than one porosome complex at the cell plasma membrane. Detailed spoke-like structures that line the porosome cup are also demonstrated for the first time in our study, providing a better understanding of the molecular physiology and architecture of this important cellular organelle. Co-isolation of porosomes with ZG demonstrated convincingly the function of the porosome in cell secretion. Furthermore, the detailed structure of the pancreatic porosome complex was revealed from our study^34^. Our current immuno-EM studies which are under way, will help determine the composition of the spokes and tethers within the pancreatic porosome complex, providing greater understanding of its composition and structure-function.

The only other permanent structure that is involved in the transport of material in cells is the nuclear pore complex, which is similar in size to the porosome complex in the exocrine pancreas or in endocrine cells, such as the growth hormone cells of the pituitary gland. The 120-125 nm nuclear pore complex is composed of nearly a 1000 protein molecules, reflecting on the complexity of the organelle similar to the porosome complex.

However, while the porosome complex is a unidirectional pore where secretory products are expelled to the outside from the cell, the nuclear pore complex is bidirectional, with proteins and nucleic acids traveling both into and from the nucleus.

- **The POROSOME is a docking station for secretory vesicles, controlling and modulating the release of their pressurized contents to the outside**.

## OPEN Questions:

1. Which SNARE proteins dictate secretory granule pools with different secretion probabilities?
2. Since maturation of immature granules is under intracellular control and is integrated into higher regulatory networks, what are the proteins that dictate it?
3. Is there a unifying control mechanism for fusion?
4. What is the biophysical meaning of the effective kinetics factor μ/λ?
