# Supplementary material for: Porosome in the Exocrine Pancreas: A Detailed EM Study suppressor
Source: Discoveries (Craiova). 2014 Aug 5;2(3):e23. doi: 10.15190/d.2014.15 (PMC6941546; doi:10.15190/d.2014.15)
Supplement: Supplementary file 1 [file discoveries-02-023-s001.pdf]

Discoveries Journals
publishing the future

DISCOVERIES 2014, Jul-Sep, 2(3): e23
DOI: 10.15190/d.2014.15

Supplementary Figures

Porosome in the Exocrine Pancreas: A Detailed EM Study

*Constantin Craciun**
Babes-Bolyai University, Cluj-Napoca, Romania

Corresponding author[*]: Constantin Craciun, Ph.D., Professor of Cell Biology, Director - Center of Electron Microscopy, Babes-Bolyai University, Cluj-Napoca, Romania;
E-mail: ccraciun@hasdeu.ubbcluj.ro;

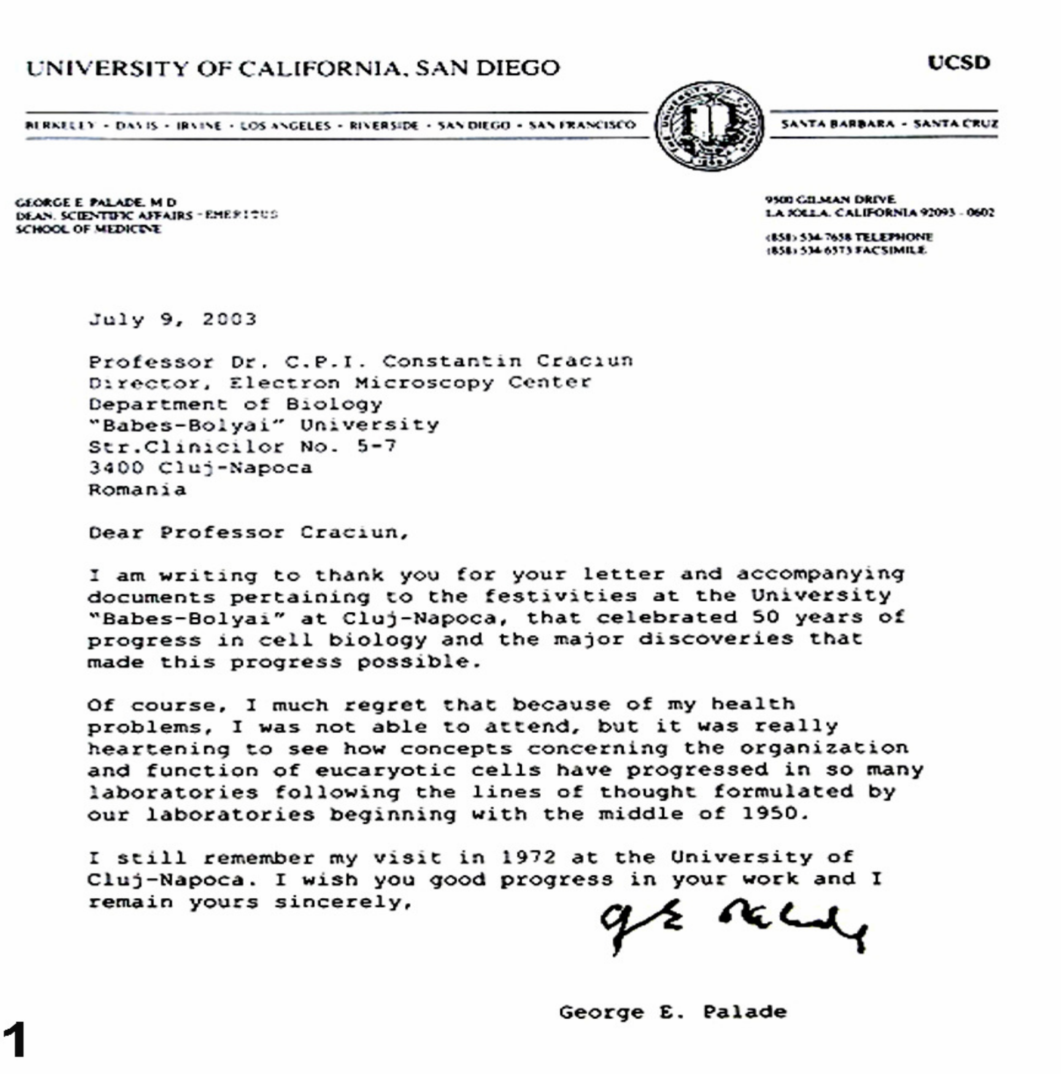

UNIVERSITY OF CALIFORNIA, SAN DIEGO — UCSD

BERKELEY • DAVIS • IRVINE • LOS ANGELES • RIVERSIDE • SAN DIEGO • SAN FRANCISCO — SANTA BARBARA • SANTA CRUZ

GEORGE E. PALADE, M.D.
DEAN, SCIENTIFIC AFFAIRS - EMERITUS
SCHOOL OF MEDICINE

9500 GILMAN DRIVE
LA JOLLA, CALIFORNIA 92093 - 0602
(858) 534-7658 TELEPHONE
(858) 534-6573 FACSIMILE

July 9, 2003

Professor Dr. C.P.I. Constantin Craciun
Director, Electron Microscopy Center
Department of Biology
"Babes-Bolyai" University
Str.Clinicilor No. 5-7
3400 Cluj-Napoca
Romania

Dear Professor Craciun,

I am writing to thank you for your letter and accompanying documents pertaining to the festivities at the University "Babes-Bolyai" at Cluj-Napoca, that celebrated 50 years of progress in cell biology and the major discoveries that made this progress possible.

Of course, I much regret that because of my health problems, I was not able to attend, but it was really heartening to see how concepts concerning the organization and function of eucaryotic cells have progressed in so many laboratories following the lines of thought formulated by our laboratories beginning with the middle of 1950.

I still remember my visit in 1972 at the University of Cluj-Napoca. I wish you good progress in your work and I remain yours sincerely,

George E. Palade

1

Supplementary Figure 1: Letter from the late Professor Dr. George E. Palade, Nobel Laureate in Physiology or Medicine, 1974
